# Outcome of urogenital infection with *Chlamydia muridarum *in CD-14 gene knockout mice

**DOI:** 10.1186/1471-2334-6-144

**Published:** 2006-09-22

**Authors:** Muhammad T Imtiaz, Justin H Schripsema, Ira M Sigar, Kyle H Ramsey

**Affiliations:** 1 Department of Microbiology and Immunology, Chicago College of Osteopathic Medicine, Midwestern University, 555 31^st ^Street, Downers Grove, IL, 60515, USA

## Abstract

**Background:**

CD14 has been postulated to play a role in chlamydial immunity and immunopathology. There is evidence to support this role in human infections but its function in a mouse model has not been investigated.

**Methods:**

Female CD14 gene knockout and C57BL/6J wild type mice were infected intravaginally with *Chlamydia muridarum*. The infection course was monitored by detection of viable chlamydiae from serially collected cervical-vaginal swabs. The sequela of tubal factor infertility was assessed using hydrosalpinx formation as a surrogate marker.

**Results:**

A significantly abbreviated infection course was observed in the CD14 gene knockout mice but hydrosalpinx formation occurred at similar rates between the two groups.

**Conclusion:**

Involvement of CD14 during chlamydial infection impedes infection resolution but this does not affect the sequela of infertility as assessed by hydrosalpinx formation.

## Background

A mouse model of urogenital chlamydial infection has been established for the study of immunity [1,2] and pathogenesis [3-7] to chlamydial infections. While in-roads have been made into understanding protective immunity in this model, much less is known about the factors associated with pathogenesis leading to the chronic sequelae of infection. One of the sequelae is tubal scarring and infertility, and a surrogate marker of infertility – hydrosalpinx formation [7,8]. Several recent reports suggest that acute inflammatory responses may cause an initial insult leading to these outcomes [7,9] but persistent infections that drive chronic inflammation are also possible causes [6].

Toll-like receptors (TLR) are a primary means of initiating innate immune system responses through the recognition of pathogen-associated molecular patterns (PAMPs) [10,11]. We read with interest the recent work of Darville, et al., that assessed the role of TLR-2 and TLR-4 in the mouse model chlamydial infection [4]. In this study, they found that although TLR-2 knockout (-/-) mice had a similar infection course as wild type (wt) controls, they showed a significant reduction in oviduct pathology and the accompanying proinflammatory cytokine production. A similar effect was not observed in TLR-4 mice. These results indicated that TLR2 is a major means of signaling to induce innate immune responses in this model.

At the time of the Darville report, our laboratory was involved in assessing the role of CD14 in the murine model. CD14 has been proved to be an accessory protein for TLR4 recognition of lipopolysaccharide (LPS) and subsequent transduction of intracellular signaling via nuclear factor-kappaB [12]. While the LPS of chlamydia has been shown to have relatively weak endotoxic activity [13], we thought it was possible that CD14 could also function as a critical accessory protein for other PAMPs such as heat shock proteins (HSP) [14] or for the removal of apoptotic cells by macrophages – an event which could regulate inflammatory responses [5,15-17]. In humans, reports have shown that a functional polymorphism in the CD14 gene is not involved in susceptibility to *C. trachomatis *infection or fallopian tube pathology [18]. Nonetheless, similar functional polymorphisms have been variously associated with susceptibility to *C. pneumoniae *infection and possibly pathological immune responses [19-22]. Based on these observations, we hypothesized that CD14 would be involved in chlamydial pathogenesis in the mouse model through one of these means or by heretofore unrecognized pathways.

## Methods

### Mice

We acquired CD14-/- breeder pairs on a C57BL/6J background (Jackson Laboratories, Bar Harbor, Maine) and initiated a colony at the Midwestern University Animal Resource Facility. CD14 gene deletion was confirmed by extracting genomic DNA from tail snips or ear punches of breeders and randomly selected offspring and assessing the presence or absence of the neomycin resistance insert and the CD14 gene by previously described methods [23,24]. All mice were housed and maintained in accordance with institutional and federal guidelines and each protocol involved in the experiments detailed below was approved by the Midwestern University Research and Animal Care Committee.

### Infection and infection assessment

At 6–10 weeks of age, female CD14-/- mice and C57BL/6J wild type (wt) controls (Harlan Sprague Dawley, Indianapolis, Indiana) were pretreated with progesterone (2.5 mg subcutaneously) and infected one week later with 100 ID_50 _of MoPn (10^4 ^IFU Weiss strain) according to previously described methods [25]. At 4, 7, 10, and 14 days post-infection and every 7 days thereafter, we collected cervical-vaginal swabs for culture of MoPn in HeLa 229 monolayer and inclusion-forming units (IFU) were visualized using indirect fluorescent microscopy and quantitated at each time point [26]. The experiments were repeated once with a total of 18 animals assessed in each group for both experiments.

### Assessment of gross pathology

Mice were euthanized and necropsied on day 56 post-infection according to previously described methods [26]. This is a time frame chosen to represent 2 to 3 weeks beyond the last culture-positive in all groups. Hydrosalpinx, an enlarged distended oviduct filled with a clear serous fluid, is a chronic sequela of infection that is a surrogate marker of infertility and indicates fibrotic tubal occlusion [7]. It is readily observed by the unaided eye in most incidences but all mice were additionally observed at 10 × magnification to ensure accuracy of results and that minor hydrosalpinx was not missed. Each mouse was graded as 0 (no hydrosalpinx), 1 (unilateral hydrosalpinx), or 2 (bilateral hydrosalpinx) and the results recorded for 2 experiments. The total number of mice assessed for hydrosalpinx for both experiments was 21 for C57BL/6J mice and 22 for CD14-/- mice.

### Statistics

A Kruskal-Wallis analysis of the variance (variables were animal, day post-infection and group) was used to assess differences in infection course between the CD14-/- mice and C57BL/6 wt mice. McNemar's chi square analysis with Yates correction was used to determine differences in culture-positive to culture-negative and in gross pathology between CD14-/- mice and the C57BL/6 controls.

## Results

### CD14-/- mice resolve chlamydial urogenital infection more quickly than wild type mice

**Figure 1:**
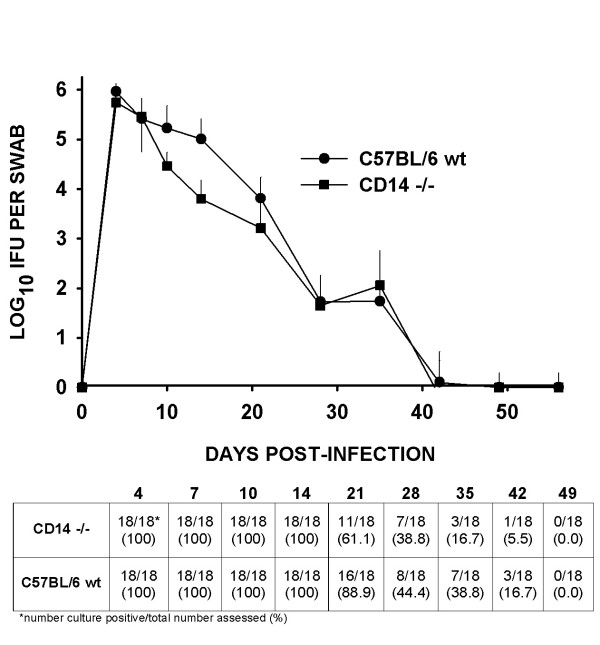
Course of *C. muridarum *(MoPn) infection in CD14-/- and wt C57BL/6J mice as assessed by quantitation of viable organisms shed from the urogenital tract. Mouse pneumonitis IFUs were isolated from cervical-vaginal swabs collected at 4, 7, 10 and 14 days and every 7 days thereafter through day 49. Each data point represents the mean (symbol) and standard deviation (vertical line) of IFU observed in HeLa 229 cultures of cervical-vaginal swab material collected from only the culture-positive mice as labeled by group in the chart at that time point based on a Log_10 _scale. Also, the ratio of culture-positive results out of the total number of mice assessed at each time point is expressed in the table below the chart. A significantly lower IFU count was observed in the CD14-/- group when the course of infection overall was compared by a Kruskal-Wallis analysis of the variance (*P *< 0.00001). In the table below the chart, by 21 days post-infection more mice resolved infection (culture-negative) beginning day 21 post-infection in the CD14-/- group (P = 0.04, McNemar's chi square analysis with Yates' correction).

Figure 1 shows the consolidated results of IFU counts over time for two experiments on a Log_10 _scale. The lines for wt C57BL/6J and the CD14-/- mice overlapped on day 4 and 7 post-infection. However, by day 10 and 14, there was a trend toward lower IFU recovery in the CD14-/- group. On day 10, the mean IFU count for CD14-/- mice was approximately 85% lower than that of C57BL/6J (1.7 × 10^5 ^+/- 3.1 × 10^5 ^IFU in C57BL/6J mice versus 2.9 × 10^4 ^+/- 2.8 × 10^4 ^IFU in the CD14-/- mice). At 14 days post-infection, a 94% lower IFU count was recorded for CD14-/- mice (6.3 × 10^3 ^+/- 8.9 × 10^3 ^IFU) when compared to C57BL/6J mice (1.1 × 10^5 ^+/- 1.6 × 10^5 ^IFU). By 21 days post-infection the mean IFU of 11 remaining culture-positive mice out 18 in the CD14-/- group (2.6 × 10^3 ^+/- 7.9 × 10^3^) and 16 remaining culture-positive mice out of 18 in the C57BL/6J wt group (7.3 × 10^3 ^+/- 1.1 × 10^4^) was only 65% different. Overall, the infection course as assessed by IFU count in CD14-/- mice was significantly abbreviated when assessed by a Kruskal-Wallis analysis of the variance (*P *< 0.00001).

Also, when we assessed the number of animals culture positive of the total assessed at each time point (see table at the bottom of Figure 1), we found that significantly more animals had resolved infection by day 21 in the CD14-/- group (7 of 18 resolved) than in the wt C57BL/6 (only 2 of 18 resolved) controls (*P *= 0.04, McNemar's chi square analysis with Yates correction). A similar trend toward fewer culture-positive mice in CD14 was observed at 28 through 42 days post-infection but this did not prove significant.

### CD14-/- mice display similar rates of hydrosalpinx formation as wild type mice

**Figure 2:**
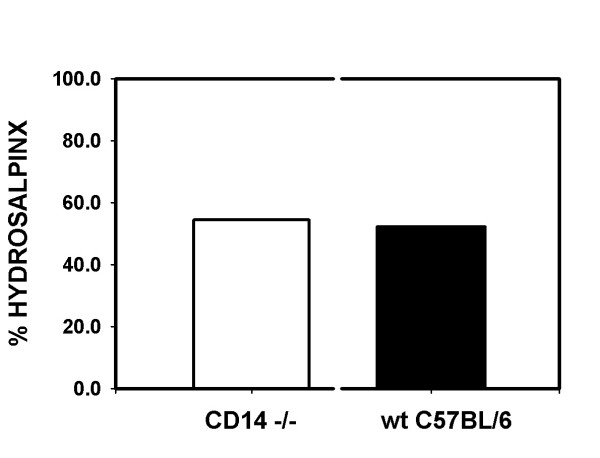
Rate of hydrosalpinx formation at day 56 post-infection in CD14-/- and wt C57BL/6J mice. The results for 44 oviducts assessed (22 mice) from CD14-/- mice are shown in the white bar whereas the results of 42 oviducts (21 mice) from wt C57BL/6 Jmice are shown in the black bar. The results are the composite of 2 separate experiments. There is no significant difference between the two groups (P = 0.8774, McNemar's chi square analysis with Yates correction).

Figure 2 depicts the rates of hydrosalpinx formation at 56 days post-infection in wt C57BL/6J and CD14-/- mice. We found a similar rate of hydrosalpinx between the two groups with CD14-/- mice displaying a 54.5% rate of hydrosalpinx formation (24 of 44 oviducts assessed) and a 52.4% (22 of 42) rate in the wt C57BL/6J group (P = 0.8774, McNemar's chi square analysis with Yates correction). When considered with Figure 1, we conclude that though the infection course is somewhat abbreviated as a result of CD14 deletion, this has no effect on chronic sequelae, and by extension, the degree of damage inflicted on the tissue by the innate immune response.

## Discussion

Along with LPS-binding protein (LBP) and MD-2, CD14 has been proved to be an accessory protein for TLR4 recognition of lipopolysaccharide (LPS) and subsequent transduction of intracellular signaling via nuclear factor-kappaB for proinflammatory cytokine production [12]. While the LPS of chlamydia has been shown to have relatively weak endotoxic activity [13], we thought it possible that a high concentration of chlamydial LPS at the foci of an infection, could serve to synergize with other PAMPs to initiate or influence inflammatory responses. Other possible chlamydial PAMP that could engage CD14 exist. For example, it has been shown that CD14 binds heat shock proteins (hsp) [14] and the chlamydial hsp has been implicated as a hypersensitizing antigen at least partly responsible for the pathogenesis of *C. trachomatis *infections [27,28]. Also, regulation of apoptosis in the murine model has been proved to have profound effects on immunopathology [5,15] and CD14 has been shown to serve as receptor on macrophages for the recognition of cells undergoing apoptosis [16,17]. In other models of Gram-negative bacterial infection, CD14 gene knockout mice had been proved to have altered outcomes of infection. Examples of these include *Acinetobacter bauminii *[29], *Legionella pnuemophila *[30], *Borrelia burdorferi *[31] and *Salmonella typhimurium *[32].

Several recent reports indicate that the *Chlamydiae *can engage TLR through LPS and non-LPS-dependent pathways [13,20,28]. However, TLR2, and not TLR4 appears to be important for the initial signaling pathway for proinflammatory cytokine production during chlamydial infection [20,33]. Most relevant to the present findings, Darville, et al., have recently shown a lack of involvement of the TLR4 but a prominent role for TLR2 in the MoPn model of female urogenital chlamydial infection and in murine fibroblasts and macrophages [4]. TLR2-/- mice and primary cell cultures produced lower levels of proinflammatory chemokines and cytokines, displayed significantly lower pathology scores and a lower rate of hydrosalpinx formation when compared to wt control mice. TLR4-/- mice showed no significant change in infection course or pathology but some increases in cytokine production were observed compared to wt control mice and primary cell lines derived from these [34].

Our present results showing a lack of involvement of CD14 in pathogenesis largely agree with the work of the Darville report regarding TLR4. We make this correlation with Darville's findings because CD-14 is an integral component TLR4 signaling pathway. In this regard, it functions as a co-receptor for LPS [12], HSP [14], and apoptotic cells for phagocytic clearance by macrophages [17]. Some divergence with the findings of Darville, et al. was found in the observation that there was a statistically significant abbreviation of infection course in CD14-/- mice, particularly during the time frame of onset of the adaptive immune response and infection resolution (days 10–21 post-infection) [35]. Though the reason for this observation remains unclear, it could be that signaling through CD14-TLR4 pathway induces a proinflammatory cytokine profile that protracts resolution of the infection by impeding the appropriate adaptive immune response. A complete and exhaustive study of cellular or antibody-mediated immune responses would be required to substantiate this hypothesis.

It should also be noted that the MoPn agent is a natural pathogen for the mouse and sustains a significantly more vigorous infection, a far higher incidence of ascending upper genital tract infection, and higher acute inflammatory responses than human *C. trachomatis *serovars in the mouse [3]. Perhaps, a strain difference in chlamydial PAMP expression or in host expression of TLRs could account for this. In this respect, the application of our results to human infections, as with all animal models, should be carefully interpreted.

Another caveat for interpreting our results relates to our use of progesterone pretreatment. This is used to enhance infection by halting the rapid estrus cycle of the mouse and providing a lush epithelial environment for growth of this pathogen that prefers epithelial cells for replication. Without such treatment, we have found that a one hundred percent infection rate cannot be obtained unless mice are inoculated on 2 or more consecutive days with much higher doses of the organism. While we can find no reports in the literature related to progesterone modulating CD14 expression, we cannot completely rule out that pretreatment with progesterone could have blurred our results.

Due to the required intracellular replication of the *Chlamydiae*, it is more likely that intracellular pattern recognition receptors are involved in initiating host responses. To this end, the aforementioned work by Darville, et al. [4] and more recent findings of Welter-Stahl, et al., suggest that this may be the case [36]. These findings indicate that intracellular pattern recognition receptors such as Nod1 and TLR2, rather than those found extracellularly, are likely candidates. While PAMP such as a rudimentary peptidoglycan expressed by the *Chlamydiae *may contribute to the initiation of the host response, certainly other chlamydial ligands are involved. These findings complicate vaccine development that targets neutralization of chlamydial PAMPs in order to prevent harmful inflammatory responses because restriction of expression of TLR agonists to the intracellular niche protects these antigens from neutralizing antibodies.

## Conclusion

The results herein imply that engagement of CD14 in this model does not affect chronic disease progression (e.g. hydrosalpinx formation and by extension, infertility) but does impede resolution of the infection.

## Abbreviations

ANOVA – analysis of variance

IFU(s) – inclusion forming unit(s)

HSP(s) – heat shock protein(s)

LPS – lipopolysaccharide

MoPn – mouse pneumonitis

PAMP(s) – pathogen associated molecular pattern(s)

TLR(s) – toll-like receptor(s)

## Competing interests

The author(s) declare that they have no competing interests.

## Authors' contributions

All authors have read and approved the final manuscript.

MTI assisted in the design of the experiment, conducted *in vivo *work, to include infection and sample collection, drafted the manuscript and conducted data compilation.

J.H.S. Assisted in the conduct of the *in vivo *work and sample collection, was responsible for CD14-/- mouse breeding and maintenance and manuscript review.

I.M.S. Conducted the culture of chlamydia for infection, isolation and quantitation of chlamydiae from cervical vaginal swabs, knockout verification and manuscript review.

K.H.R. Was overall responsible for the implementation and design of the of the experiments, assisted with sample collection and statistical analysis and was responsible for the final preparation and drafting of the manuscript for submission.

## Pre-publication history

The pre-publication history for this paper can be accessed here:


